# A 38-year study on *Trichinella* spp. in wild boar (*Sus scrofa*) of Latvia shows a stable incidence with an increased parasite biomass in the last decade

**DOI:** 10.1186/s13071-015-0753-1

**Published:** 2015-03-01

**Authors:** Muza Kirjušina, Gunita Deksne, Gianluca Marucci, Eduards Bakasejevs, Inese Jahundoviča, Anžela Daukšte, Aleksandra Zdankovska, Zanda Bērziņa, Zanda Esīte, Antonino Bella, Fabio Galati, Angelika Krūmiņa, Edoardo Pozio

**Affiliations:** Daugavpils University, Institute of Life Sciences and Technology, Daugavpils, Latvia; Institute of Food Safety, Animal Health and Environment “BIOR”, Riga, Latvia; European Union Reference Laboratory for Parasites, Istituto Superiore di Sanità, Rome, Italy; CNESPS, Istituto Superiore di Sanità, Rome, Italy; SIDBAE, Istituto Superiore di Sanità, Rome, Italy; Riga Stradiņš University, Riga, Latvia

**Keywords:** *Trichinella britovi*, Latvia, Wild boar, *Sus scrofa*, Incidence, Epidemiology, Biomass, Snow cover, Carnivore

## Abstract

**Background:**

*Trichinella* spp. are zoonotic parasites transmitted to humans by the consumption of raw or insufficiently cooked meat of different animal species. The most common source of infection for humans is meat from pigs and wild boar (*Sus scrofa*). The aim of the present work was to evaluate the incidence of *Trichinella* spp. infections in wild boar hunted in Latvia over a 38 year interval (1976 to 2013).

**Methods:**

A total 120,609 wild boars were individually tested for *Trichinella* spp. by trichinoscopy and, in case of negativity, by artificial digestion of 25 g muscles, in the 1976–2005 period, and by artificial digestion of 25–50 g muscles in the 2006–2013 period. *Trichinella* spp. larvae were identified at the species level by multiplex PCR.

**Results:**

In the study period, the overall prevalence of infected wild boar was 2.5%. *Trichinella britovi* was the predominant (90%) species. The incidence of *Trichinella* spp. infection in wild boar exhibited two different trends. From 1976 to 1987, the incidence of infected/hunted wild boar increased from 0.23% to 2.56%, then it decreased to 0.19 in 1994. Thereafter, the incidence fluctuated between 0.05% and 0.37%. A statistically significant (*P* < 0.05) correlation (r = 0.54; p = 0.0199) was found between the trend of *Trichinella* spp. incidence in hunted wild boar and the number of snow cover days from 1976 to 1993. From 1997 to 2013, the estimated wild boar population of Latvia increased by 4.9 times and the hunting bag by 9.7 times, with a stable incidence of *Trichinella* spp. in the population. It follows that the biomass of *Trichinella* spp. larvae and of *T. britovi*, in particular, increased.

**Conclusions:**

The incidence trends of *Trichinella* spp. in wild boar could be related to the role played by the snow in reducing the thermal shock and muscle putrefaction which increases the survival of the larvae in muscle tissues of carrion in the 1976–1993 period; and, in the 1997–2013 period, to the increased biomass of *Trichinella* spp. due to the increased carnivore populations, which are the main reservoirs of these parasites.

## Background

Nematodes of the genus *Trichinella* are cosmopolitan parasites of carnivorous and omnivorous animals, which can be transmitted to humans by ingestion of raw or semi-raw meat and meat products of different animal origin [1]. The main source of infection for humans is meat from domestic pigs and wild boar (*Sus scrofa*) [2].

In Latvia, *Trichinella* spp. infections have been documented in wolves, wild boar and humans since 1960 [3]. From 1955 to 1985, trichinellosis was documented in 152 people who had acquired the infection for the consumption of meat, mostly from wild boar [4]. From 1986 to 2000, 150 cases of human trichinellosis due to the consumption of smoked or undercooked pork (72%), wild boar meat (23%), and unknown sources (5%), were gathered from [5]. Since 1992, human trichinellosis increased from 0.1-0.2 (1982–1991) to 3.75 (2000) cases per 100,000 inhabitants [6]. Then in the period 2000–2009, the average incidence of trichinellosis was 1.1 per 100,000 inhabitants of Latvia [2]. In the last fifteen years, there has been a marked reduction of human trichinellosis caused by the consumption of pork from domestic pigs in most countries of the European Union. In contrast, the number of infections caused by the consumption of meat from hunted wild boar has remained stable [2]. Among carnivore mammals of Latvia, *Trichinella* spp. has been detected in the lynx (*Lynx lynx*), red fox (*Vulpes vulpes*), raccoon dog (*Nyctereutes procyonoides*), wolf (*Canis lupus*), and pine marten (*Martes martes*) [7-10].

The aims of the present work were to evaluate by a retrospective analysis of longitudinal data, the relationship between the *Trichinella* spp. incidence in Latvian wild boar and its population growth during the past 38 years, to explore the cause(s), which could have affected the *Trichinella* spp. incidence in the wild boar population, and to identify the *Trichinella* species circulating in Latvian wildlife.

## Methods

### Source of information

The number of hunted wild boar tested for *Trichinella* spp. infection and the number of positive wild boar per district per year, were collected from the annual reports of the National Veterinary Laboratory for the period 1976–1992, from the annual reports of the Veterinary Medicine Diagnostic Centre for the period 1992–2006, and from the annual reports of the National Diagnostic Centre and of the Institute of Food Safety, Animal Health and Environment BIOR, for the period 2006–2013. Wild boars were collected from all the 26 districts (according to the previous administrative division) of Latvia throughout the year. Data on the estimated wild boar population on April 1^st^ of each year from 1976 to 1990, was gathered from [11]. For the period 1991–2013, this information was downloaded from [12].

Data on the estimated raccoon dog and red fox populations were from [11] and from the State Forest Service (Jānis Ozoliņš, personal communication) for 2010. From 2005 to 2011, carnivore mammals (lynxes, raccoon dogs, red foxes, wolves, martens, domestic dogs, and domestic cats) hunted or killed by cars in Latvia were also screened to detect *Trichinella* spp. infection and to identify the etiological agents.

### Detection of *Trichinella* infection

From 1968 to 2005 according to the Latvian Normative act Nr. 5-I0-960 of Latvian SSR Ministry of Agriculture of 9 October 1968, hunters delivered to veterinary services on a voluntary basis no less than 100 g of diaphragm muscle and 50 g of tongue muscle from hunted wild boar. *Trichinella* spp. larvae were searched in 28 small pieces about the size of a grain of rice from the diaphragm samples, by trichinoscopy. When trichinoscopy was negative, 25 g of muscle samples were individually digested by artificial gastric juice [13]. Briefly, 25 g from diaphragm and/or tongue muscles of each animal were cut into small pieces by scissors. Chopped meat was then placed on a bee sieve of 15 cm, which was placed in turn on a funnel containing the digestion fluid (1000 ml of warm water, 5 g of pepsin, 7 ml of HCl) covering the meat. The meat was incubated at 39°C for 18 h. Then 5 ml of the digestion fluid was run off from the bottom of the funnel in a conical tube. After 30 min sedimentation, 3 ml of supernatant were discharged and the remaining 2 ml were poured out in a 6 cm Petri dish. Larvae were searched under a stereomicroscope at 40× magnifications. The laboratory personnel were regularly trained on trichinoscopy and digestion methods with frequent observations of positive samples. When a positive wild boar was detected, the carcass was burned down in the presence of a veterinary inspector. From 1976 to 2005, the larval burden per gram of muscle was not evaluated and larvae were not collected for their identification at the species level.

From 2006 to 2013, muscle samples from wild boar (25–50 g) and carnivores (25 g) were individually tested by the magnetic stirrer method according to the Commission Regulation 2075/2005 (European Commission, 2005) [14]. The larval burden per gram of muscle was not evaluated but larvae were collected, washed in PBS, and then stored in 70%-96% ethyl alcohol for their identification at the species level by a molecular test. Information on *Trichinella* spp. isolates is available at the website of the International Trichinella Reference Centre [15].

### Molecular identification of *Trichinella* spp. larvae

From 2005 to 2013, at least five single *Trichinella* spp. larvae isolated by artificial digestion from each positive animal, were identified at the species level by multiplex PCR analysis according to previously published protocols [16,17].

### Potential factors influencing the *Trichinella* spp. prevalence in wild boar

The following factors were examined to evaluate their potential influence on the *Trichinella* spp. incidence in wild boar of Latvia in the period 1976–2013: 1) the number of *Trichinella* spp. infecting domestic pigs per year (Annual reports from 1976 to 1992 of the National Veterinary Laboratory; Annual reports from 1992 to 2006 of the Veterinary Medicine Diagnostic Centre; Annual reports from 2006 to 2011 of the National Diagnostic Centre and of the Institute of Food Safety, Animal Health and Environment BIOR), assuming the *Trichinella* spp. transmission from the domestic to the sylvatic cycle; 2) the estimated carnivore populations (red foxes and raccoon dogs) per year, since their carcasses left by hunters on the ground, can be the source of *Trichinella* spp. infections for wild boar; 3) the number of hunted wild boar per year for the reason cited in point 2; 4) the estimated wild boar population per year, since an increased population can result in a feed shortage favouring scavenging behaviour on carrions; 5) the air temperature and precipitation per year [18,19]; and 6) the number of snow cover days gathered from [20] for the period 1976–2004, and from the website (ftp://ftp.ncdc.noaa.gov/pub/data/gsod) for the period 2005–2013.

### Statistical analysis

The proportion of infected wild boar was evaluated by the Chi-square for trend test. The prevalence of *Trichinella* spp. was calculated by dividing the number of infected wild boar by the number of hunted wild boar × 100. The Pearson’s Correlation Coefficient was used to compare the number of days with snow cover and the rate *Trichinella* sp. infected/hunted wild boar. P < 0.05 was considered significant. The statistical analysis was performed using the STATA 11.2 software.

## Results

**Figure 1:**
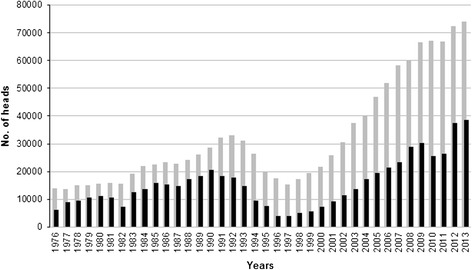
**Estimated and hunted wild boar heads in Latvia from 1976 to 2013.** Number of estimated heads, grey bar; number of hunted heads, black bar.

**Figure 2:**
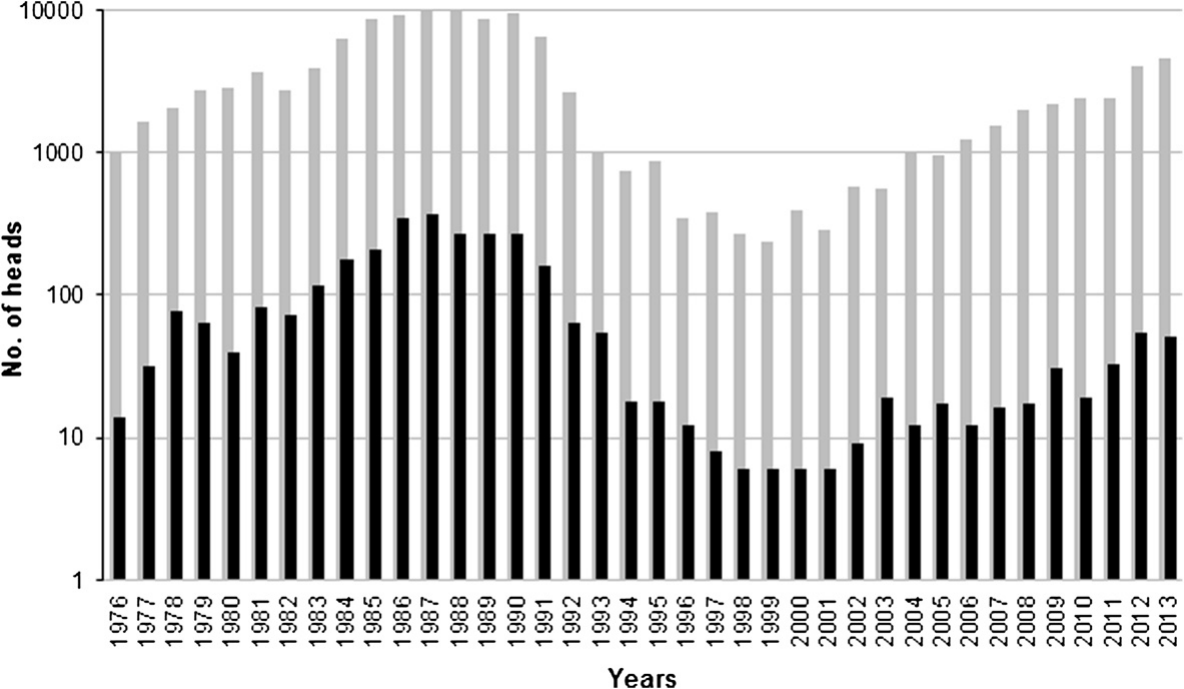
**Tested wild boar and** ***Trichinella*** **spp. positive wild boar in Latvia from 1976 to 2013.** Number of animals tested for *Trichinella* spp., grey bar; number of *Trichinella* spp. positive animals, black bar. Logarithmic scale.

**Figure 3:**
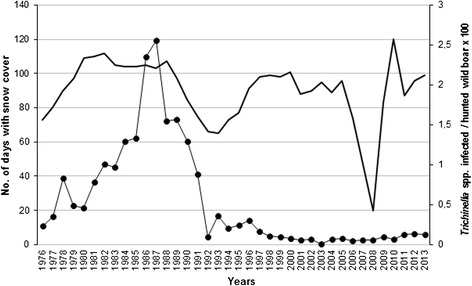
**Prevalence of** ***Trichinella*** **spp. in hunted wild boar and snow cover days in Latvia.** Number of snow cover days from 1976 to 2013, solid line; *Trichinella* spp. infected/hunted wild boar × 100, line with black circles.

In the 38-year period (1976–2013), the estimated average number of wild boar in Latvia was 32,244 heads (range 13,775-74,107) per year (Table 1). The estimated wild boar population size fluctuated in the study period reaching a peak of 33,039 heads in 1992 and a new peak of 74,107 heads in 2013 (Figure 1). The number of hunted heads followed a similar fluctuation (average 15,496; range 3,962-38,723) and was proportional to the estimated wild boar population size (average 48%; range 22.5% - 72.5%) (Figure 1). From 1997 to 2013, the estimated wild boar population increased by 4.9 times and the hunting bag increased by 9.7 times. An average of 20% (range 0.54% - 60.9%) of the hunted wild boar were tested for *Trichinella* spp. and an overall prevalence of 2.5% was detected in the study period (Table 1). The number of tested wild boar and the number of *Trichinella* spp. positive animals varied among the years (Table 1, Figure 2). From 1976 to 1987, the incidence of infected/hunted wild boar increased from 0.23% to 2.56%, then it decreased to 0.19 in 1994. Thereafter, the incidence fluctuated between 0.05% and 0.37% (Figure 3).

**Table 1:** **Estimated, hunted, tested and** ***Trichinella*** **spp. positive wild boar (** ***Sus scrofa*** **) in Latvia**

	**Average per year (range)**	**%**
Estimated wild boar population	32,244 (13,775-74,107)	
Hunted wild boar	15,496 (3,962-38,723)	48^a^
Wild boar tested for *Trichinella* spp.	3,174 (238–10,138)	20^b^
Wild boar positive for *Trichinella* spp.	80 (6–369)	2.5^c^

Data have been collected from 1976 to 2013.

^a^on estimated wild boar population.

^b^on hunted wild boar.

^c^on tested wild boar.

A statistically significant correlation (r = 0.54; p = 0.0199) was found between the trend of *Trichinella* spp. incidence in wild boar and the number of snow cover days from 1976 to 1993 (Figure 3). In contrast, from 1994 to 2013, no correlation (r = 0.08; p = 0.7628) was observed between these two variables.

**Figure 4:**
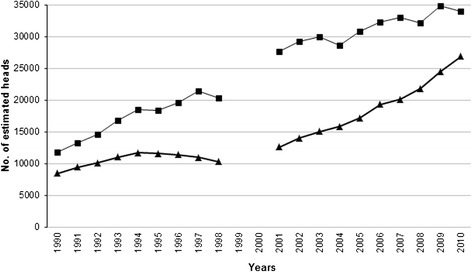
**Yearly estimate of red fox and raccoon dog heads in Latvia from 1990 to 2010.** Red fox, line with boxes; raccoon dog, line with triangles. No data is available for the years 1999–2000.

From 1990 to 2010, the estimated population size of raccoon dogs and red foxes increased by 288% and 317%, respectively (Figure 4). A correlation was detected between the increased carnivore populations and increased *Trichinella* spp. biomass in the wild boar population. No relationship was observed between the other investigated variables and the trend of *Trichinella* spp. incidence in the wild boar population (data not shown).

**Figure 5:**
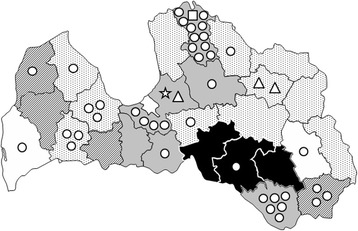
**Prevalence of** ***Trichinella*** **spp. in wild boar by Latvian district from 1976 to 2013.** White districts, prevalence ≤ 1; dotted districts, prevalence > 1 ≤ 2; stripped districts, prevalence > 2 ≤ 3; grey districts, prevalence > 3 ≤ 4; black districts, prevalence > 4. *Trichinella* spp. isolates from wild boar: *Trichinella britovi*, circles; *Trichinella nativa*, triangles; *Trichinella spiralis* star; mixed *T. britovi*/*T. nativa*, box. The district origin of seven *T. britovi* isolates from wild boar is unknown.

No correlation was detected between the incidence of *Trichinella* spp. infection in wild boar and their geographical origin, based on the 26 Latvian districts nor on the four main Latvian regions (Kurzeme, Latgale, Vidzeme and Zemgale) (data not shown). The overall prevalence for the 38 year period ranged from 0.8% in the Rezekne district up to 10.1% in the Preili district with the highest prevalence in the centrum and south-eastern districts of Latvia (Figure 5).

*Trichinella britovi* was recovered from 90% of the *Trichinella* spp. isolates from wild boar hunted in 15 districts (Figure 5). *Trichinella nativa* was isolated from three (5.8%) wild boars killed in two districts; *T. spiralis* was detected in one (1.9%) wild boar and a mixed *T. britovi*/*T. nativa* infection in another wild boar (Figure 5).

Overall, from 2005 to 2011, *Trichinella* spp. larvae were isolated from 179 carnivores. Larvae were identified as *T. britovi* (96%), *T. nativa* (2.8%) and *T. britovi*/*T. nativa* mixed infections (1.1%). *Trichinella britovi* was detected in 50 foxes, 43 lynxes, 43 raccoon dogs, 33 wolves, 1 marten, 1 domestic dog and 1 domestic cat; *T. nativa* in 3 wolves and 2 foxes; and mixed *T. britovi*/*T. nativa* infections in 1 wolf and 1 fox.

## Discussion

We present valuable data here on the long term (38 years) trends in the incidence of *Trichinella* spp. in the hunted wild boar population of Latvia. They reveal an increase from 0.23% to 2.56% in the period 1976–1987, a decrease up to 0.19% in 1994, and then a fluctuation from 0.05% to 0.37% in the following years (Figure 3). This trend was not linked to the observed 4.9 fold growth of the host population in the country, with an average number of 1.14 heads per square kilometre in 2013.

A correlation (r = 0.54; p = 0.0199) between the incidence trend of *Trichinella* spp. in wild boar and the number of snow cover day trend during the period 1976–1993 has been observed in the present study (Figure 3). The higher the number of snow cover days, the higher the *Trichinella* spp. incidence in wild boar and vice versa. This suggests that snowfall favours the survival of *Trichinella* spp. larvae in decaying host muscles by preventing sudden changes of the carrion temperature (e.g., the action of the wind), and by maintaining a constant humidity. This correlation stresses the importance of the time spent by the larvae in the decaying muscle tissues when they are no longer protected by the host homeothermy. The muscle larvae have an anaerobic metabolism which supports their survival in decaying muscles over long periods of time, even though the striated muscle tissue develops an angiogenesis process around the muscle cell-larva complex [21]. Madsen [22] considered the ecological niche of the host carrion as the environment of the “free-living” stage, equivalent to the egg stage of most other nematode species. However, in the absence of snow cover, the carcass can be exposed to rapid decrease/increase of temperature, causing freezing and/or thawing of the muscle tissues which kill the larvae, and/or to a fast drying of the muscle tissues also resulting in the death of the larvae. However, further investigations are needed to evaluate the influence of the environment under the snow [23] on the survival of *Trichinella* spp. larvae in the host carcasses.

Since the *Trichinella* spp. incidence in the wild boar population of Latvia was quite uniform in the last 12 years (Figure 3), we can assume a significant increase of the parasite biomass in wild boar due to the growth of wild boar and carnivore populations (Figures 1 and 4). In Latvia, this increased parasite biomass is represented for 90% by *T. britovi* even if this species is not considered to be well adapted to swine (see below).

*Trichinella britovi* is largely the predominant *Trichinella* species circulating in Latvia where, in addition to wild boar, its prevalence in wild carnivores may vary from 21% to 50% in the raccoon dog, 17%-57% in the red fox, 69.6% in the wolf, 40%-90% in the lynx, and 46.1% in the pine marten [7-10]. *Trichinella spiralis* has been documented only in six foxes and in four domestic pigs [8], and in one wild boar (present work).

The enteral and parenteral niches of swine are not very favourable to *T. britovi*; a low worm burden and a short survival time in the muscles is typical [24-26]. Therefore, the overall prevalence of 2.5% in wild boar during the period 1976–2013 (1.4% in the last 12 years) should be considered very high. In European countries, where *T. britovi* is the prevalent species in wildlife, the prevalence of infection in wild boar is 0.3 in Estonia [8], 0.007 in Hungary [27], from 0.002 to 0.017 in Italy [28], 0.51 in Lithuania [8], and 0.06 in the Slovak Republic [29].

Carnivore mammals, canids in particular, are the most important reservoir species of *T. britovi* and *T. nativa* [30,31]. In some European countries, the increase of *Trichinella* spp. prevalence among wild boar has been related to a concomitant increase of the carnivore populations. In Poland, an increase in the fox population density has been linked with an increase in the prevalence of *Trichinella* spp. among wild boar [32,33]. In Mecklenburg–Western Pomerania (Germany), the increase of the *Trichinella* spp. prevalence in wild boar has been associated with the increasing raccoon dog population in the region [34]. In Spain, a decreasing trend of *Trichinella* spp. in the wild boar population during 1998 to 2009, was associated with an increase in fenced areas, which prevented the circulation of wild carnivores considered the most important reservoir of *Trichinella* spp. [35]. In Latvia, there has been a concomitant increase of the wild boar and carnivore populations. In 2010, the total estimated number of wild carnivores (lynx, raccoon dog, red fox, wolf, American mink, badger, pine and stone martens, and polecat, about 140,000 heads) [11], the main reservoir hosts for *T. britovi* [31], was two times higher than the number of estimated wild boar heads (about 67,000, Figure 1). The abundance of carnivores in which the prevalence of *Trichinella* spp. (96% *T. britovi*) was extremely high, allows the maintenance of a stable incidence of infection in the wild boar population in spite a 4.9 fold increase and the short survival time of *T. britovi* in this host species.

The increase of the carnivore and wild boar population size may have enhanced the common habit of hunters to leave animal carcasses in the field after skinning, or removing and discarding the entrails, which has been demonstrated to strongly increase the probability of *Trichinella* spp. transmission among wildlife [22,36-42], and to free-ranging and backyard pigs, particularly if the pig owner is a hunter [42].

In the period 1976–2005, the use of the trichinoscopy test in first instance and then, in the case of a negative result, of the digestion test of single animals, was driven to reduce the number of apparatuses needed to test these animals. The pooled sample digestion test was not used to reduce the need to identify the positive animal/s present in a pool considering an expected prevalence up to 40% in some districts (data not shown). The use of two, at least in part different, artificial digestion protocols between the 1976–2005 and 2006–2013 periods, does not seem to have influenced the test sensibility, since the incidence detected in the last eight years (2006–2013) was similar to the incidence of the previous eight years (1998–2005).

## Conclusions

In most European countries including Latvia, but also elsewhere, the number of wild boar tested for *Trichinella* spp. larvae is always a percentage of the hunted animals (Figures 1 and 2). A high percentage of these animals are hunted for own consumption, do not enter into the official market, and escape the veterinary controls, thus causing infections in humans [43]. There is the need to educate hunters on the importance of the systematic examination for *Trichinella* spp. larvae of game intended for human consumption to prevent human infection. Furthermore, veterinary services should educate hunters not to spread game carcasses or their scraps and offal in the environment, and should organize a system for a proper collection and disposal of these biological samples.
